# Competition Strategies of Metritic and Healthy Transition Cows

**DOI:** 10.3390/ani10050854

**Published:** 2020-05-15

**Authors:** Borbala Foris, Marina A. G. von Keyserlingk, Daniel M. Weary

**Affiliations:** Animal Welfare Program, Faculty of Land and Food Systems, University of British Columbia, Vancouver, BC V6T 1Z4, Canada; forisb@mail.ubc.ca (B.F.); nina@mail.ubc.ca (M.A.G.v.K.)

**Keywords:** welfare, coping strategy, precision livestock farming, sickness behavior

## Abstract

**Simple Summary:**

Competition for feed is a social stressor for dairy cows and is associated with an increased risk of illness. We investigated how cows trade off the motivation to feed together with group mates against the risk of competitive interactions at the feeder, and in this way identified each individual’s competition strategy. We then related these strategies to cow health. Competition strategies varied between cows and showed low to moderate stability over time. Strategies of metritic and healthy cows did not differ before or after calving, but metritic cows changed strategies more upon entering the social group after calving, particularly in the days before diagnosis. We conclude that cows show individual competition strategies, and that automated measures of strategy change may help in detecting metritis.

**Abstract:**

Our study aimed to characterize social competition strategies in transition cows, and determine how these varied with health status. We retrospectively followed 52 cows during 3 periods (PRE: d −6 to −1 prepartum, POST1: d 1 to 3 postpartum, POST2: d 4 to 6 postpartum). Cows diagnosed with metritis on d 6 postpartum (*n* = 26) were match paired with healthy cows (*n* = 26). Measures of agonistic behavior (i.e., replacements at the feeder) and feeding synchrony were determined by an algorithm based on electronic feed bin data, and used to calculate competition strategies via principal component analysis. We found consistent strategies, defined by two components (asynchrony and competitiveness; explaining 82% of the total variance). We observed no differences in strategies when comparing healthy and metritic cows, but metritic cows tended to change their strategies more between PRE and POST1, and between POST1 and POST2, indicating that strategies change in association with parturition and metritis. We conclude that cows show individual variation in competition strategies, and that automated measures of strategy change may help in detecting metritis.

## 1. Introduction

There is growing evidence that social behavior during the transition period can predict the risk of illness [1,2,3]. Regrouping and competition for resources such as feed are two common stressors [4,5]. Cows are motivated to feed together with conspecifics [6,7], meaning that behavioral synchrony impacts feeding behavior, which in turn affects competition at the feed bunk when feed availability or feeding space is limited [8]. Some have argued that increased feeding synchrony indicates reduced competitiveness and positive welfare, especially for lower ranking cows [7,9,10]. However, in indoor housing systems, agonistic interactions at the feed bunk are frequent [11], especially when the feeding space is limited [12]. These results suggest that there is a tradeoff between the motivation to engage in synchronized feeding and the risk of agonistic interactions.

There is considerable individual variation in feeding behavior (reviewed in [13]), much of which cannot be explained by social rank [14,15]. Competition at the feed bunk has been described in terms of coping [16,17], or other social strategies [15,18,19], which describe consistent individual behavioral responses to competition for feed. Differences in social competition strategies employed by cows may reflect how they trade off feeding synchrony versus agonistic behavior. Specifically, some cows may feed at peak times, despite having to engage in agonistic interactions to gain access to feed (a proactive strategy), and others may choose to feed at non-peak times (i.e., when few groupmates are feeding) to reduce the risk of agonistic interactions (a reactive strategy).

Changes in agonistic and feeding behavior can be used to identify animals at risk of becoming ill [20,21,22]. Prepartum feeding time, intake, and agonistic interactions at the feed bunk are related to the risk of postpartum disease [23,24,25] (but see also [26]). Moreover, living in an unpredictable social environment increases both the number of agonistic interactions and the risk of uterine disease in multiparous cows [27]. Individuals may adjust their social competition strategy according to changes in external conditions, internal states, and individual differences in behavioral plasticity [28,29]. We predicted that changes in individual social competition strategies during the transition period would be associated with metritis, based on two key pieces of evidence. Firstly, some cows may be more susceptible to changes in the social or physical environment and cannot maintain consistent feeding behavior [30,31], which can exacerbate the effects of negative energy balance [17]. Second, social competition strategies may change around the onset of clinical symptoms, as a type of sickness behavior [21,32,33].

Automated measures of feeding behavior can be used to assess social competition. In this study, we used validated algorithms based on electronic feed and water bin data to record agonistic replacements [34,35,36]. Our aims were to characterize social competition strategies based on measures of agonistic behavior and behavioral synchrony at the feed bunk, to test if healthy and metritic cows exhibit different strategies, and to determine if these strategies change during the transition period.

## 2. Materials and Methods

Cows enrolled in this study were a subsample of animals reported in previous work investigating behavioral changes before metritis diagnosis [26], the effects of nonsteroidal anti-inflammatory drug treatment [37], and the association between metritis and behavior in the lying stall [38]. All cows were housed at The University of British Columbia’s Dairy Education and Research Center (Agassiz, British Columbia, Canada), and they were cared for under the Canadian Council of Animal Care guidelines [39]. The study was approved by the UBC Animal Ethics Committee (Protocol A14–0040).

For a detailed description of housing conditions, procedures, and diet, see [26]. Briefly, pre- and postpartum cows were housed in pens equipped with 24 lying stalls, 12 Insentec (Insentec, Marknesse, The Netherlands) feed bins and 2 Insentec water bins. Cows were moved into the prepartum pen 3 weeks before calving. Upon signs of calving, they were moved to the maternity pen, where a maximum of 2 cows were kept at any given time. Within 24 h after calving, cows were introduced into the postpartum group, where they stayed for another 3 weeks. Group composition was dynamic, but stocking density was maintained at 20 cows per pen. Pre- and postpartum groups were fed fresh TMR [26] formulated according to their nutritional needs [40] at approximately 0800 and 1600 h, and postpartum cows were milked twice per day, at approximately 0700 and 1700 h. In the postpartum group cow health was monitored for 21 d; cows were screened for metritis every 3 d after the morning milking and metritis was diagnosed based on vaginal discharge [23,41].

We included cows in our Metritic group if they were diagnosed as healthy on d 3 and metritic on d 6 postpartum, and if they had no other clinical disease or evidence of subclinical ketosis (blood BHB < 1.2 mmol/L). Cows that did not develop any clinical health disorder, did not have subclinical ketosis, showed no more than 1 day of fever (rectal temperature ≥ 39.5 °C) d1 to d6 postpartum, were included in our Healthy group. In total, 52 cows were enrolled, 26 metritic cows (10 multiparous and 16 primiparous) were match paired with 26 healthy cows, based on closest in calving date to the paired metritic cow, balancing for parity, and body weight.

Electronic feed and water bin data from all cows, beginning 6 d before calving and ending 6 d after calving, were divided into 3 periods: (1) PRE; d −6 d to d −1; (2) POST1; d 1 to d 3; (3) POST2; d 4 to d 6. The behavioral parameters (Table 1; the complete data set, description, and analysis files are provided as Supplementary Material) in each of the 3 observation periods were derived from the electronic bin data as follows. An algorithm (validated in [34]) was used to detect agonistic replacements, defined as when one cow competitively displaced another from a feed bin and occupied the same bin within 26 s. Data from feed and water bins were combined and filtered for false positive replacements, following [36]. For each cow, the number of actor and reactor replacements was determined. Water bin data were not considered, given the low number of replacements detected, and will not be discussed further. Behavioral synchrony, defined as performing the same behavior as the majority of group members at the same time [42], was not biologically meaningful in the context of the present study in which cows were overstocked at the feed bins (12 bins for 20 cows). Therefore, to measure individual-level feeding synchrony, we calculated the total number of occupied feed bins for each second when a focal cow was present at any of the feed bins. In addition, we determined the number of free bins at the time of each actor and reactor replacement, and calculated total daily feeding time.

All analyses were performed in R, version 3.5.3 [43]. The level of significance was set to *p* < 0.05 and 0.05 ≤ *p* < 0.1 was considered as a trend. For each cow, we calculated the mean daily values within each period for all behavioral parameters, resulting in 3 measurements (PRE, POST1, POST2) for 6 behaviors (Table 1).

To determine the main components of social competition at the feed bunk, we performed a principal component analysis (PCA) with varimax rotation, using the R package psych [44]. Behavioral parameters were investigated for normality using the Shapiro–Wilk test. Not all parameters were normally distributed, therefore, the Spearman correlation matrix was used as input to the PCA. We performed a PCA on the 6 behavioral parameters measured during the PRE period. The applicability of PCA was evaluated using the Kaiser–Mayer–Olkin criterion (MSA = 0.55), and 2 rotated components (RC) were extracted based on eigenvalues and the Horn parallel test [45]. We then calculated RC scores for cows using the weights obtained and the standardized behavioral parameters. The same weights were used with POST1 and POST2 measurements to obtain RC scores for these periods. To evaluate if the components represented consistent behavioral strategies, we calculated the stability between periods using the Pearson correlation, and the Benjamini–Hochberg method [46] was used to correct the *p*-values for multiple testing.

Using the RC scores, we investigated if healthy and metritic cows differed in social competition strategies during PRE, POST1, and POST2 periods. First, a linear model was used to evaluate the effect of metritis on RC scores in each observation period, while controlling for parity. Metritis (metritic (*n* = 26), healthy (*n* = 26)), parity (primiparous (*n* = 16), multiparous (*n* = 10)) and their interaction were considered fixed effects. The fixed effects were retained and the interaction term was dropped from the final model if there was no effect. Second, we investigated if healthy and metritic cows differed in how much their social competition strategy changed between periods. We considered the strategy change associated with moving to the postpartum group (PRE-POST1) and with the onset of metritis (POST1-POST2) separately. For each cow, the social competition strategy was determined by the 2 RC scores, resulting in one point in the two-dimensional space for each period. To quantify the magnitude of strategy changes, we calculated the Euclidian distance between PRE and POST1, as well as POST1 and POST2 points. The effect of metritis on the magnitude of strategy changes was tested using the same model as described above.

## 3. Results

Descriptive statistics for the recorded behavioral parameters are reported in Table S1.

### 3.1. Characterizing Social Competition Strategies

PCA resulted in a two-dimensional model of social competition strategies, explaining 82% of the total variance in the data (Table 2). Components were named based on the parameters with the strongest loadings [47]. RC1 was labelled asynchrony, due to a strong negative loading for synchrony and strong positive loadings for the number of free bins during actor and reactor replacements. RC2 was labelled competitiveness, due to strong positive loadings for displacements (as both actor and reactor) and feeding time.

RC scores showed a positive association across periods for both components. For competitiveness correlations of moderate strength (*r* range: 0.63–0.71, *p* < 0.001) were obtained across all 3 periods; correlations for asynchrony were weaker (*r* range: 0.27–0.43, *p* < 0.05).

### 3.2. Association Between Metritis and Social Competition Strategies

**Figure 1:**
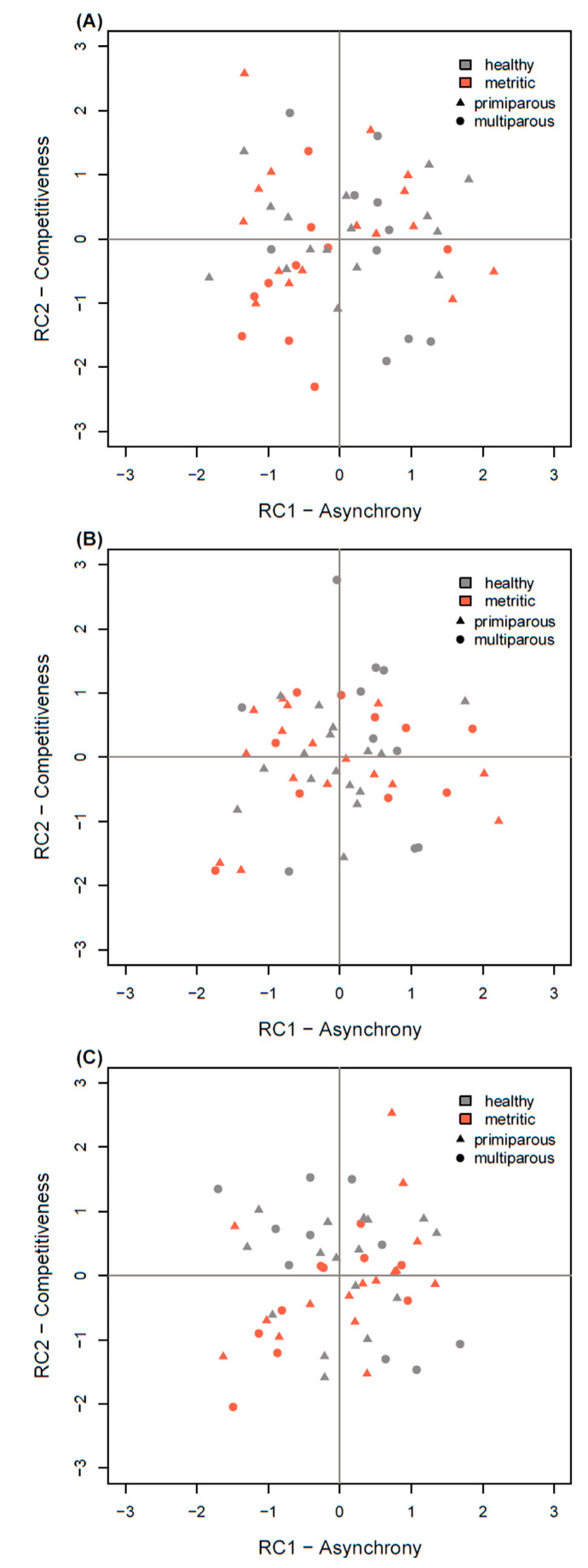
Principal component (RC) scores of healthy (*n* = 26) and metritic (*n* = 26) cows in 3 different periods (**A**): PRE: d −6 to −1 prepartum, (**B**): POST1: d 1 to 3 postpartum, (**C**): POST2: d 4 to 6 postpartum). Metritic cows were healthy during PRE and POST1 and diagnosed with metritis on d 6 postpartum. The RC scores of healthy and metritic cows did not show significant differences in any period, but primiparous cows tended to have higher RC2 scores during PRE.

The social competition strategies of cows, as determined by the combined RC scores, are shown in Figure 1. Linear models did not show differences in the RC scores of healthy and metritic cows in any period. We did, however, note a parity effect on RC2 scores in the PRE period, where primiparous cows tended to be more competitive (F_1,49_ = 3.39, *p* = 0.072).

**Figure 2:**
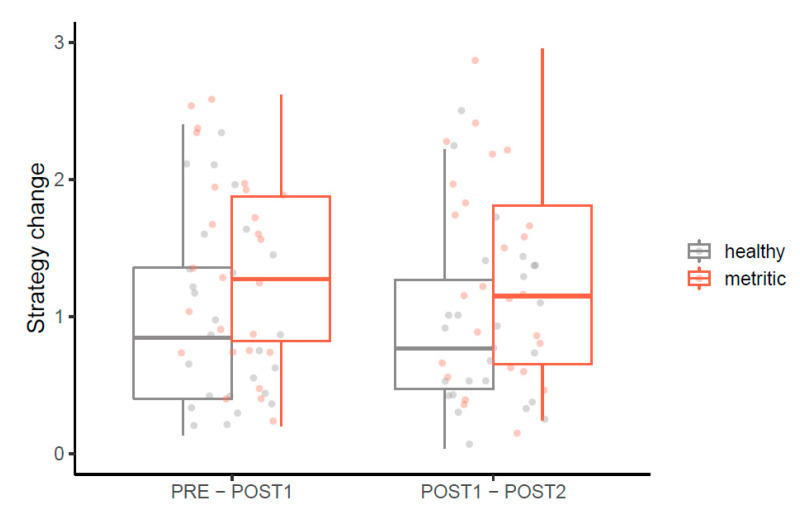
Magnitude of change in competition strategies between observation periods (PRE: d −6 to −1 prepartum, POST1: d 1 to 3 postpartum, POST2: d 4 to 6 postpartum) for healthy (*n* = 26) and metritic (*n* = 26) cows. Competition strategies are defined in 2-dimensional space by asynchrony and competitiveness scores; the *y*-axis shows strategy change between observation periods, calculated as the distance between corresponding points. Compared to healthy cows, metritic cows showed a greater change in competition strategy from PRE to POST1 (*p* = 0.06) and from POST1 to POST2 (*p* = 0.04).

The magnitude of individual change in social competition strategies, corresponding to moving to the postpartum group (i.e., Euclidian distance between PRE-POST1 RC scores), and to the onset of metritis (i.e., Euclidian distance between POST1-POST2 RC scores) is presented in Figure 2. Metritic cows tended to change their social competition strategies more than healthy cows when they entered the postpartum group (F_1,49_ = 3.67, *p* = 0.06). Moreover, metritic cows showed a greater change in strategy between POST1 and POST2 periods (F_1,49_ = 4.46, *p* = 0.04). The direction of change varied between individuals (Figure S1) and parity did not relate to strategy change.

## 4. Discussion

Our results indicate that individual variability in social competition during the transition period can be characterized by measures of agonistic behavior and feeding synchrony. To date, most studies have relied on the use of single behavioral parameters to investigate the association between social competition and illness, e.g., [27,48]. To our knowledge, ours is the first study using electronic feed bins to detect feeding synchrony during the transition period. We measured the level of competition, taking into consideration the number of free bins available for each agonistic replacement to better account for the difficulty in gaining access to food. In line with previous work [14], replacements were observed even when few cows were feeding indicating that social competition can be motivated by factors in addition to access to food.

The PCA analysis revealed two main factors, asynchrony and competitiveness. As indicated by the parameters with high loadings, cows with high asynchrony scores had lower feeding synchrony, and the majority of their agonistic interactions took place when many feed bins were unoccupied. High competitiveness scores corresponded to longer feeding times and more agonistic interactions, both as actor and reactor. Both components showed a positive association across observation periods, indicating that they correspond to social competition strategies, which are consistent over time. This result is in line with previous work describing consistent individual differences in feeding and social behavior during the weeks before calving [49]. Competitiveness showed a stronger association between periods than asynchrony, perhaps because when a cow is introduced to a new group, familiarization and behavioral synchronization take more than 6 d to develop [50]. Social bonds might also have been a contributing factor, as these bonds are known to influence physical proximity and behavioral synchrony [51,52]. In addition, feeding synchrony may have been affected by milking during the postpartum period, as milking is associated with time away from the pen.

As expected, the competition strategies that cows expressed are consistent with previously described coping styles [16,53]. Cows with high competitiveness and low asynchrony (i.e., engaging in many replacements, feeding at peak times) could be described as proactive copers; and cows showing low competitiveness and high asynchrony (i.e., avoiding replacements, feeding at non-peak times) could be described as reactive copers. Some cows, however, exhibited a strategy characterized by high asynchrony and high competitiveness (i.e., engaging in replacements, but mostly at non-peak times), and others had low asynchrony and low competitiveness scores (i.e., feeding at peak times, but with few replacements). These cows may represent an intermediate coping style, or more subtle proactive strategies [16,54]. Replacements at non-peak times might also correspond to individual variability in exploratory behavior [55], and aggressiveness, and differences in these traits may explain the variability within proactive copers [56]. Our observation that primiparous cows tend to show higher competitiveness during the prepartum period may reflect that they were less dominant [57] and thus replaced many times, in line with previous findings that primiparous cows are replaced more often due to their lower body weight [58].

Our results underline the role of individual variation during the transition period, a topic that has recently gained interest (e.g., [49]). Understanding variation in social competition strategies may help improve management on farms. For instance, cows that initiate agonistic interactions during non-peak times might consistently bully others, posing a challenge for cows that shift feeding times to avoid agonistic interactions. In contrast, low competitiveness and asynchrony may allow cows to feed at peak times and avoid most agonistic interactions. We encourage future research to determine how social competition strategies can be accounted for when creating or modifying cow groups.

An association between coping style and immune function has long been suggested [16,56,59]. However, we failed to find a difference in component scores between metritic and healthy cows, implying that social competition strategy, per se, is neither a risk factor nor an indicator of metritis. Previous work in our lab found that cows diagnosed with metritis and subclinical ketosis postpartum engaged in fewer agonistic interactions at the feed bunk in the week before calving and consumed less feed [23,24,26], and these differences were more pronounced when cows had multiple health disorders [25]. In the current study, we focused only on metritis, but future work could investigate the change in social competition strategies for cows with multiple illnesses. Koolhaas et al. [16] proposed that animals with a reactive coping style are more flexible in their behavior and thus perform better in unpredictable environments, and that predictable environments favor proactive ones that are generally less flexible. A recent study on transition dairy cows found that the relationship between feeding behavior and endometritis depended upon the predictability and competitiveness of the social environment [49]. Therefore, it is possible that group composition (in terms of social competition strategies of group members) modulates the relationship between individual social competition strategy and illness.

According to game theory, the success of any one strategy will depend upon its frequency as well as the frequency of other strategies used by members in the group [60]; a proactive strategy will be especially successful if many others adopt a passive strategy, and vice versa. Hence, whether a given strategy is helpful to the animal (including reducing their risk of illness) will vary depending upon the mix of strategies used in the group [61]. Such group-to-group differences may account for the lack of relationship between social competition strategy and metritis in our study.

The costs and benefits of implementing a strategy depend on the state (e.g., energy reserve, health status) of the individual, and changes in state may be associated with a change in strategy [62,63,64]. In our study, cows that developed metritis changed their strategies more than those that remained healthy. The change in strategy may have related to the early onset of malaise and rise in sickness behaviors during the days before diagnosis [17]. Moreover, when introduced to a new social group, the competition strategy of some cows may change, based on the effectiveness of this strategy relative to the strategies used by the other group members [60]; if changing strategies adds to the social stress experienced by the cow, and if this increased stress puts cows at increased risk of metritis, this may explain the association between metritis and strategy change in the current study.

In our study, the group composition changed dynamically which may have limited the establishment of a stable social hierarchy and thus lead to more agonistic interactions. Future work investigating how social competition strategies of individuals change when moving cows from one stable group to another would complement our results. The methods used to assess social competition strategies provide opportunities for future research focused on monitoring changes in behavior and social dominance over time [13,65]. We used electronic bins primarily suited for research facilities, but similar parameters may be recorded with simpler devices [66]. In future work, measuring how cows modify competition strategies when the strategies of other group members change may aid in the detection of individuals having difficulty coping. Automated measures of competitiveness and feeding synchrony may help inform how changes in group composition affect social stress.

## 5. Conclusions

Automated measures of agonistic behavior and feeding synchrony can be used to identify consistent social competition strategies, defined by different levels of asynchrony and competitiveness. Metritis was not directly related to the competition strategy, but compared to healthy cows, metritic cows tended to change their strategies more in the first three days after entering the postpartum group, and in the three days around diagnosis. A better understanding of individual variation in competition strategies may help in transition group management. Moreover, precision farming technologies may enable the automated measurement of strategy change, potentially facilitating the early detection of metritis.

## Figures and Tables

**Table 1:** Definitions of the behaviors recorded. All measures were recorded daily for each cow.

Parameter	Definition
Feeding time	Total time a cow spent at the feed bins (s)
Synchrony	Mean no. of occupied bins when the cow was feeding
Actor	No. of times a cow was the actor in a replacement at a feed bin
Reactor	No. of times a cow was the reactor in a replacement at a feed bin
Free bins when actor	Mean no. of free bins during actor replacements
Free bins when reactor	Mean no. of free bins during reactor replacements

**Table 2:** Loadings matrix of the Principal Component Analysis performed on behavioral measures in the prepartum period, derived from electronic feeders. Eigenvalues and proportions of total variation explained by each component (RC) are reported. Components were named based on loadings > 0.70, indicated with bold.

Parameter	RC1	RC2
Feeding time	0.19	**0.88**
Synchrony	**−0.89**	0.10
Actor	−0.48	**0.73**
Reactor	−0.40	**0.76**
Free bins when actor	**0.92**	−0.15
Free bins when reactor	**0.95**	−0.15
Eigenvalue	3.45	1.45
Variance explained	49.4%	32.3%
Component name	Asynchrony	Competitiveness

## Supplementary Materials

The following are available online at https://www.mdpi.com/2076-2615/10/5/854/s1. Figure S1: Two-dimensional change in social competition strategies between observation periods, Table S1: Descriptive statistics of behavioral parameters recorded in three periods, Supplementary Data 1: Daily measures of six behavioral parameters, based on electronic feed bin data, metritis, and parity information from 52 cows, Supplementary File 1: Description for Supplementary Data 1, Supplementary File 2: R script for statistical analysis.
