# Supplementary material for: Competition Strategies of Metritic and Healthy Transition Cows
Source: Animals (Basel). 2020 May 15;10(5):854. doi: 10.3390/ani10050854 (PMC7278391; doi:10.3390/ani10050854)
Supplement: Supplementary file 1 [file animals-10-00854-s001.zip › animals-781537-suppl_BF.docx]

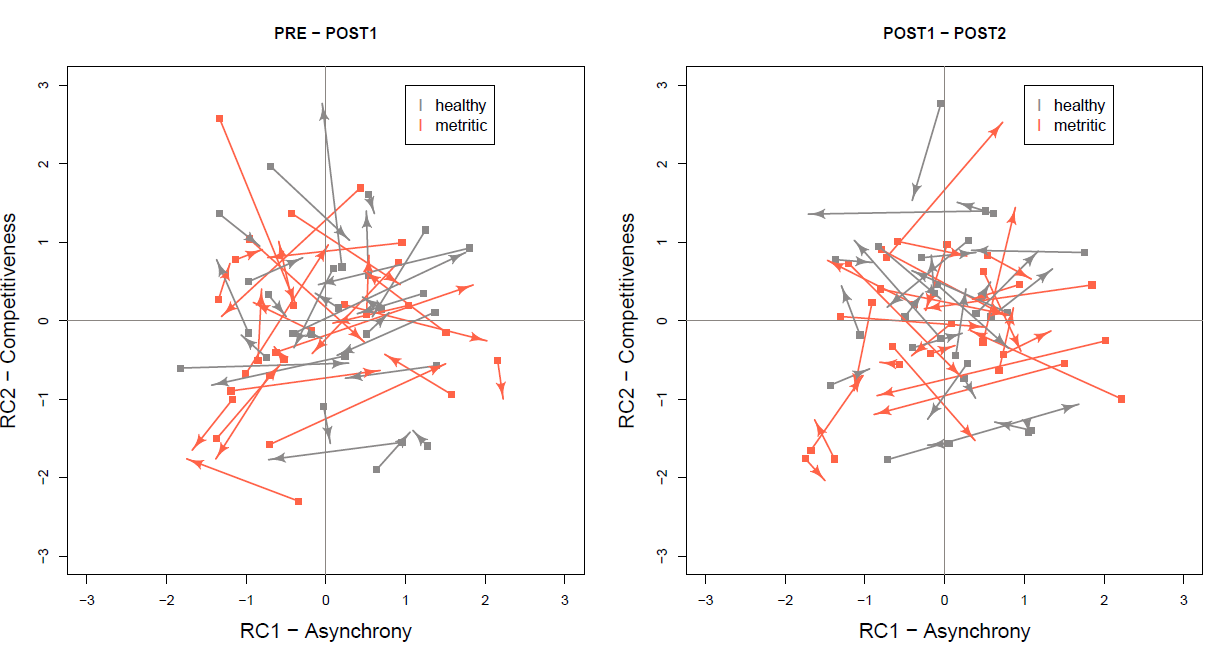


**Figure S1.** Two-dimensional change in social competition strategies between observation periods (PRE: d ₋6 to ₋1 prepartum, POST1: d 1 to 3 postpartum, POST2: d 4 to 6 postpartum), for healthy (n = 26) and metritic (n = 26) cows. Social competition strategies are defined by asynchrony and competitiveness scores; arrows point from the first measurement to the second.

**Table S1.** Descriptive statistics of behavioral parameters recorded in three periods (PRE: d ₋6 to ₋1 prepartum, POST1: d 1 to 3 postpartum, POST2: d 4 to 6 postpartum), based on electronic feed bin data from 52 cows.

| **Parameter** | **Mean** | **SD** | **Min** | **Max** |
| --- | --- | --- | --- | --- |
| **PRE** |  |  |  |  |
| Feeding time (s) | 13918.04 | 2733.45 | 7825.17 | 18855.17 |
| Synchrony | 6.34 | 0.82 | 4.51 | 7.80 |
| Actor | 34.28 | 15.73 | 7.00 | 86.33 |
| Reactor | 34.47 | 17.24 | 3.00 | 80.83 |
| Free bins when actor | 4.46 | 1.10 | 2.45 | 6.73 |
| Free bins when reactor | 4.40 | 1.03 | 2.08 | 6.57 |
| **POST1** |  |  |  |  |
| Feeding time (s) | 9352.55 | 1947.46 | 5186.33 | 12934.00 |
| Synchrony | 5.17 | 0.57 | 3.81 | 6.45 |
| Actor | 11.08 | 5.04 | 2.67 | 21.00 |
| Reactor | 12.15 | 4.75 | 3.33 | 29.67 |
| Free bins when actor | 5.07 | 1.22 | 3.22 | 8.31 |
| Free bins when reactor | 5.38 | 1.06 | 3.41 | 7.57 |
| **POST2** |  |  |  |  |
| Feeding time (s) | 10580.31 | 1812.72 | 7043.33 | 15783.33 |
| Synchrony | 5.17 | 0.49 | 4.21 | 6.45 |
| Actor | 11.91 | 5.56 | 4.00 | 23.67 |
| Reactor | 14.10 | 5.23 | 1.33 | 24.00 |
| Free bins when actor | 4.82 | 0.98 | 2.48 | 7.14 |
| Free bins when reactor | 5.21 | 0.88 | 3.29 | 7.67 |
