# Supplementary material for: Competition Strategies of Metritic and Healthy Transition Cows
Source: Animals (Basel). 2020 May 15;10(5):854. doi: 10.3390/ani10050854 (PMC7278391; doi:10.3390/ani10050854)
Supplement: Supplementary file 1 [file animals-10-00854-s001.zip › Supplementary_File1.docx]

**Table S2 - Competition Strategies of Metritic and Healthy Transition Cows**

The ***Foris_TableS2.csv*** file contains the raw data used for statistical analysis.

Columns in this table represent the following variables:

**cows:** individual cow ID

**sick:** 0 represents healthy and 1 represents metritic cows

**parity:** parity as continuous variable

**parity2:** parity as binary variable, 0 represents primiparous and 1 represents multiparous cows

**days:** number of the observation day relative to calving

**sync:** mean no. of occupied bins when the cow was feeding

**feed_time:** total time a cow spent at the feed bins (s)

**actor_feed:** no. of times a cow was the actor in a replacement at a feed bin

**mean_free_actor_feed:** mean no. of free bins during actor replacements

**receiver_feed:** no. of times a cow was the reactor in a replacement at a feed bin

**mean_free_receiver_feed:** mean no. of free bins during reactor replacements

The code for generating the results and figures in the manuscript is provided in the ***Analysis_Competition_strategies.R*** file.
